# Sinonasal Tract Malignancies: Prognostic Factors and Surgery Outcomes

**DOI:** 10.5812/ircmj.14118

**Published:** 2013-12-05

**Authors:** Abdurrahman Bugra Cengiz, Melek Uyar, Ela Comert, Engin Dursun, Adil Eryilmaz

**Affiliations:** 1 Department of Otorhinolaryngology, Oncology Hospital, Ankara, Turkey; 2 Department of Otorhinolaryngology, Recep Tayyip Erdoğan University, Rize, Turkey; 3 Department of Otorhinolaryngology 3rd Clinic, Numune Hospital, Ankara, Turkey

**Keywords:** Paranasal Sinus Neoplasms, Surgery, Survival Rates, Prognosis

## Abstract

**Background:**

Cancers of the sinonasal region are rare and its survival rate remains poor because most of the patients are asymptomatic and diagnosed in advanced stages with surrounding important structures.

**Objectives:**

This study attempted to analyze the clinical and histological features in addition to survival and prognostic factors of surgical treatment of sinonasal cancers.

**Patients and Methods:**

A retrospective cohort study, involving 36 patients with sinonasal cancer who were treated with surgery in our hospital between 2000 and 2010, was performed. Patients were selected based on the convenience sampling. Patients treated with radiotherapy and/or chemotherapy were excluded from the analysis. Clinical symptoms and histologic findings of patients as well as malignant tumor staging and its prognosis were collected from archives.

**Results:**

We found that overall 3 and 5-year survival rates of subjects were 52.8%, and 41.6%, respectively. There was a negative correlation between the clinical stage and survival. There was a significant difference between infrastructural and suprastructural localization in 5-year survival rate (P = 0.018). In the present study, there was a strong relationship between the local control and overall survival (P < 0.01). Overall 5-year survival rate was similar in patients both in the exenterated orbit and preserved orbit (P > 0.05).

**Conclusions:**

The present study has demonstrated that clinical stage, suprastructural tumor, and the presence of tumor- positive resection margins are the most significant prognostic factors affecting local tumor control and survival. As a result of this study, these tumors should be treated in early stages by surgical margin of resection followed by adjuvant radiotherapy.

## 1. Background

Malignant neoplasms of the sinonasal are uncommon tumors that constitute less than 2% of overall malignancies, and 1.5-3% of upper respiratory tract. They are often caused by occupational or environmental exposures to certain substances (1). The most frequent location is the maxillary sinus (50-80%) with the ethmoid sinuses (2) but, due to the junction of the nasal cavities with the paranasal sinuses, determining the origin of advanced sinonasal tumors is often difficult (1, 3). The initial symptoms are vague and nonspecific and the majority of patients have advanced-stage disease by the time the diagnosis is made. Possible symptoms of these cancers include: feeling pain above or below the eyes, change in vision, blockage of one side of the nose and nasal congestion (2, 4).

Tumor stage, histological differentiation and involvement of lymph nodes have been determined to be prognostic factors (1, 5). Most malignant sinonasal tumors will require surgery to remove the cancerous tissue. They are close to vital structures such as the brain, optic nerves, and internal carotid artery; therefore, the surgery may cause significant morbidity to the patients. The endoscopic surgery needs to be performed. The term endoscopic refers to the use of small nasal endoscope that allows all of the surgeries to be performed through the nostrils, without the need for any incisions on the face. Extended surgery, in this anatomic region, does not allow the gross total tumor resection en bloc with negative margins to be required by oncologic principles. Additional treatment for cancer may include radiation therapy, chemotherapy, or both.

## 2. Objectives

The purpose of this article was to evaluate the prognostic factors, disease control and survival rates of patients with sinonasal cancer who underwent surgery alone or with radiotherapy in a single clinic.

## 3. Patients and Methods

### 3.1. Patient Selection

Patients who were consecutively admitted to our clinic and diagnosed with the sinonasal malignant cancer were retrospectively evaluated between the years 2000-2010. This study was performed in Ankara Numune Training and Research Hospital, Turkey. A total of 62 patients diagnosed with sinonasal cancer have been included in this study based on hospital data. Twenty-six of these patients were excluded from the study due to the fact that they were treated with radiotherapy alone (n = 7) or radiotherapy combined with chemotherapy (n = 19). Fifteen of these patients had distant metastasis at presentationand 11 of them were old and medically unfit.

#### 3.1.1. Inclusion Criteria

The patients whose tumor should be located in the nasal cavity or paranasal sinuses and can be resected were included in this study. Thirty-six patients (mean age 50.13 ± 11 years old; 27(75%) male, 9(25%) female) who treated by surgery primarily either alone or with radiotherapy combination and who had a minimal follow-up of five years were included. The data including the age and gender of patients; the location and T classification of the primary tumor; tumor histopathology; involvement of the adjacent structures; the treatment modalities; and survival data were collected.

#### 3.1.2. Exclusion Criteria

Patients with benign tumors, such as inverted papilloma, and palate or skin primary tumors with secondary invasion of the sinuses and nose were excluded. A major part of the excluded tumors was located in the nasopharynx or vestibulum nasi. Patients with sinonasal malignancies and unscheduled surgery, during the study, were excluded for the following reasons: distant metastasis at presentation, the presence of T4b sinonasal malignity, unresectability, patients who had medical disorders preventing the performance of surgery, elderly age and patients who do not accept surgery conducted with palliative intent.

### 3.2. Work-up and Surgical Procedures

Work-up before the treatment by surgery included a nasal endoscopic examination with incisional biopsy, computed tomography (CT) and magnetic resonance imaging (MRI) in the assessment of invasion to the adjacent sites. In our study, PET/CT scan was performed for staging before any treatment. All patients consulted to the prosthodontics impressions were taken for the provision of dentate obturator. Stages were classified according to the seventh edition of American Joint Committee on Cancer TNM classification. According to the radiological images, 44% of the patients had maxillary lateral wall involvement. In 33.3% of cases, orbital base or the ethmoidal sinuses were observed. Medial maxillectomy and/or limited excision were performed on 6 (16.7%) patients. Total maxillectomy (complete removal of the maxilla without orbital exenteration) was performed on 20 (55.6%) patients. Craniofacial resection was conducted on two patients.

The orbital invasion was diagnosed according to MRI (bone infiltration at the bottom of the orbit) in 16 patients; hence, intraoperative frozen sections used from the orbit soft tissue and radical maxillectomy including orbital exenteration were performed on eight patients. Five (13.8%) patients with clinically positive LNs and five N0 patients underwent selective neck dissection.

The criteria for postoperative radiotherapy (6000-6500 cGy) were as follows: the patients who had advanced stages (Stage III-IVa), positive surgery margins, clinically and histologically positive lymph nodes, extra capsular spread, recurrence disease. Twenty eight patients had postoperative radiotherapy (77.7%) and 11 of them had chemotherapy with radiotherapy after the surgery. Recurrent disease was verified by biopsies.

### 3.3. Follow-up and Statistical Analysis

The median follow-up took 32 months (between 3- 111 months). Patients were controlled per two months at first year, and three months intervals for the second year, and twice in a year, afterwards. We wish to estimate and compare survival rates and relative risks , based on 95% confidence and at least 80% expected power. Previous studies showed that survival rates were between 40 and 65% and standard error was reported to be 0.15 %. We estimated that, in last ten years, approximately 550 patients sinonasal cancer were treated in our country. Of these patients, 300 were treated with surgery for curative intent. Our sample size formula was N= p x q x (Z_α/2_/ E) ^2^. It would be approximately 35, or (40/1.133) that has rounded to the nearest whole number. The pathological clinical variables were statistically analyzed using SPSS software, version 15.0 for Windows (SPSS® Inc. Illinois, USA). The association of qualitative characteristics was analyzed using Pearson’s χ2 correlation test or Fisher’s exact test to evaluate the independent relationship between gender, age, histology, location of the tumor based on Ohngren’s (6) description, and negative or positive surgical margins of the histopathological specimen analysis with the risk of death of sinonasal cancer. Differences were considered statistically significant when P < 0.05. The variables that were found to be significantly different by univariate analyses were subjected to multivariate logistic regression analysis and Cox's proportional hazard model. The level of confidence was 95%, in this study. for estimating survival, Kaplan-Meier curves were used to compare the survival distributions with the log-rank test.

### 3.4. Ethics

All patients signed their informed consent after receiving information about the project. The study was approved by the Ethics Committee of Numune Training and Research Hospital, Ankara, Turkey (date: 18 January 2012 number: 05/12).

## 4. Results

The clinical data of patients are shown in Table 1. The most common initial symptom was nasal obstruction (78%) followed by facial numbness, pain or/and swelling (41.6%), oral symptoms (32.4%), epistaxis (21.6%), increase of lacrimation (2.7%) which were found in our study. The most common findings were intranasal mass (72.9%) followed by intraoral mass, facial deformity and palpable neck mass. There was no evidence of distant metastases. Squamous cell carcinomas (SCC; n = 15, 41.7%) was the most represented histological variety observed in 36 patients, followed by 6 adenoid cystic carcinomas (16.7%), 3 (8.3%) adenocarcinomas, 2 undifferentiated carcinomas (5.6%), 2 malign melanomas, 2 malign mesenchymal tumors, osteosarcoma, chondrosarcoma, malign mixed tumor hemangiopericytoma, synovial sarcoma (one each) (Table 2). 

**Table 1.** Demographic Characteristics of Patients with Sinonasal Tumors (n = 36)

	No. (%)
**Gender**	
Male	27 (75)
Female	9 (25)
**Age**	
20-40	7 (19.4)
41-60	22 (61.2)
61+	7 (19.4)
**Education**	
Illiterate	3 (8.3)
Primary	6 (16.6)
Secondary	23 (63.8)
Graduate	4 (11.1)
**Occupation**	
Wood worker	1 (2.7)
Industry worker	4 (11.1)
Other	22 (61.1)
Unemployed	9 (25)
**Marital status**	
Married	33 (91.6)
Single	3 (8.4)
**Smoking habit**	26 (72.2)

**Table 2.** The Histological Classification, Localization of the Tumor and T Stages of the Patients (n = 36)

	No. (%)
**Histological Type**	
Squamous cell carcinoma	15 (41.7)
Adenoid cystic carcinoma	6 (16.7)
Adenocarcinoma	3 (8.3)
Undifferentiated carcinoma	2 (5.6)
Other epithelial forms	3 (8.3)
Non-epithelial forms	7 (19.4)
**Localization**	
Maxillary sinus	26 (72.3)
Nasal cavity	7 (19.4)
Ethmoid sinus	3 (8.3)
**Stage**	
T1	2 (5.6)
T2	6 (16.7)
T3	15 (41.7)
T4a	13 (36.1)
N+	5 (13.8)

The vast majority of cases presented with locally advanced disease (28, 77.7%); two (5.6%) patients had stage 1 (mucosal disease), 6 (16.7%) patients stage 2 (extension to adjacent sites), 15 (41.7%) patients had stage 3 (e.g. the orbital base or the ethmoidal sinuses were affected) and 13 (36.1%) patients had stage 4 (involving orbital apex, nasopharynx etc.) at the time of diagnosis. Five patients had ipsilaterally neck disease (13.8%).

**Figure 1.**
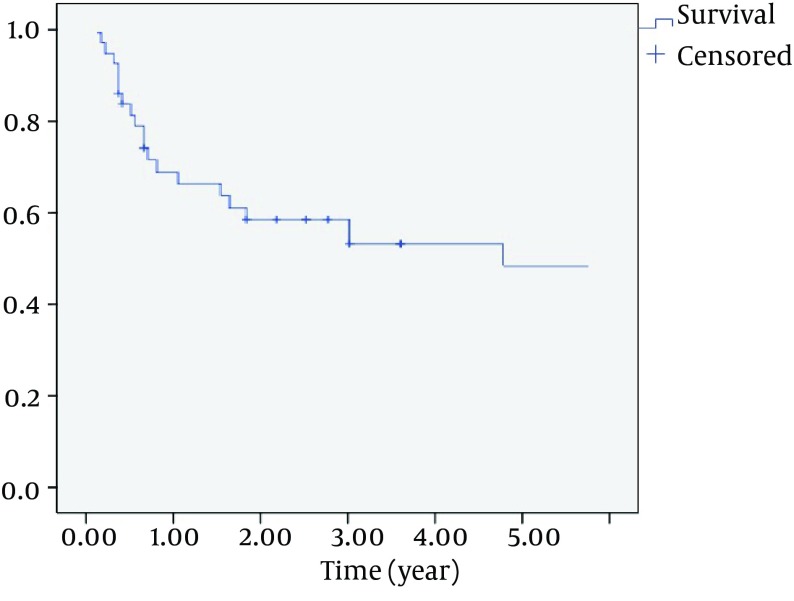
Kaplan-Meier Overall 5-year Survival Rates

**Figure 2.**
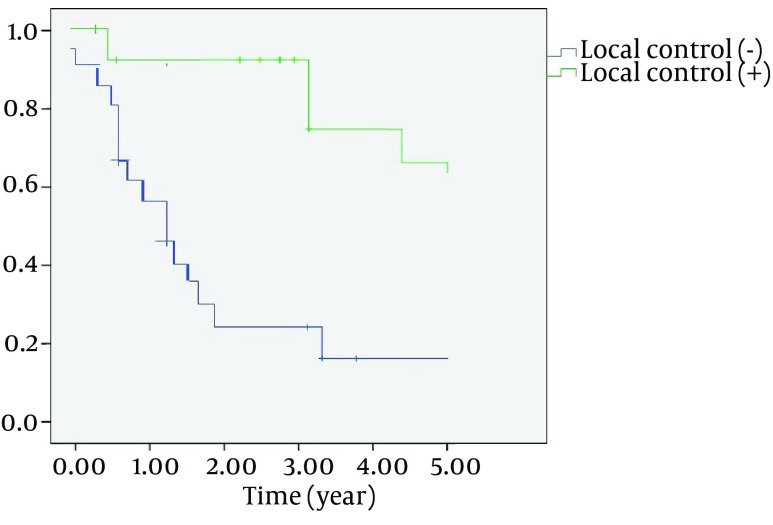
Kaplan-Meier 3-year Survival Estimated by Locoregional Failure P < 0.001 for Comparisons of Patients With (n = 22) and Without (n = 14) Locoregional Failure

The overall 3-and 5 year survival rate was 52.7% and 41.6% (Figure 1) and disease-free survival rate was 40.5% and 36.1%. Overall 3-and 5-year overall survival rate, regarding age, gender and histological type, was not statistically significant; however, undifferentiated carcinoma and sarcomas showed poor prognosis. The survival rate of 3-year suprastructure sinuses was 25%, while in infrastructure sinuses; it was 66.7% (OR = 6, CI = 1.2 - 18.4, P = 0.018) (Figure 2). 

**Figure 3.**
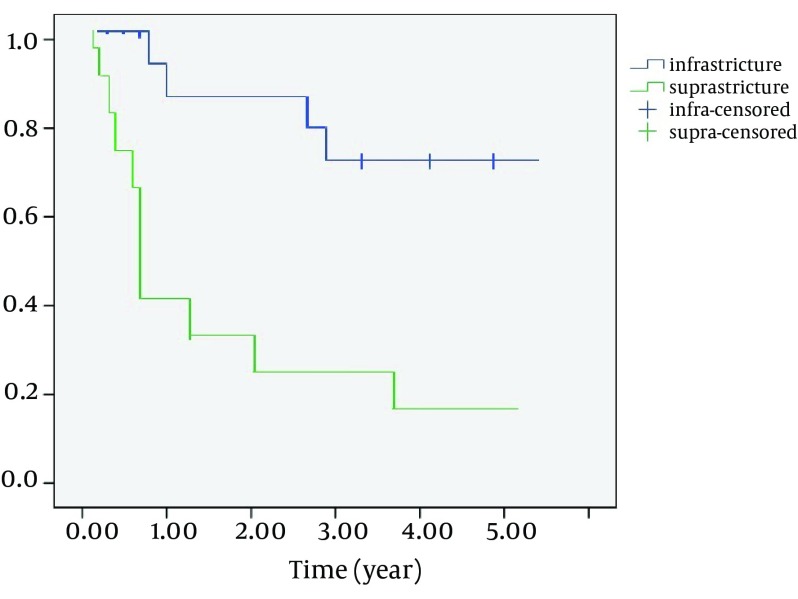
Kaplan-Meier 3-year Survival Rate Estimated by Localization of Disease. P = 0.018 for Comparisons of Patients With Suprastructure (n = 25) and Infrastructure (n = 11) Region

1-and 3- year survival rates were 82.5% and 39.3%, in early stages (I-II) and advanced stages (III-IVa), respectively (P < 0.05). Three- year survival rates were 92.9% in patients without recurrence or persistent disease and 27.3% in patients with locoregional failure. (OR = 0.2, CI = 0.03-0.371, P < 0.001) (Figure 3) After the surgery, positive surgical margin was determined in 19 (52.7%) patients according to histopathologic examination of specimen. Local recurrence was occurred in 22 patients (61.1%), postoperatively. There was a strong association between negative surgical margins and local control (Table 4). Although local control was done on only 3 (15.8 %) patients who had positive surgical margin, it also was done on 11 (64.7%) patients who had negative surgical margins (OR = 9.7, CI = 2-47.6, P = 0.005). 

**Table 3.** Main Causes of Local Control Failure and Survival Rates According to Locoregional Recurrence (P < 0.01)

	Local Control (-) (n = 22, 61.1%)	Local Control (+) (n = 14, 38.8%)	Total (n = 36)
**Positive Surgical margins, No. (%)**	16 (84.2)	3 (15.8)	19 (52.7)
**Negative Surgical margins, No. (%)**	6 (35.3)	11 (64.7)	17 (47.3)
**Stages (I-II), No. (%)**	2 (25)	6 (75)	8 (22.3)
**Stages (III-IV), No. (%)**	20 (71.4)	8 (28.6)	28 (77.7)
**3 year survival, (%)**	(27.3)	(92.9)	(52.8)
**Stage IV 3 year survival, (%)**	(11.1)	(40.5)	(30.8)

There was no statistical significance according to overall survival rate in patients who had orbit preservative surgery (37.5%) or orbital exenteration (25%) (Table 4) (P > 0.05). Distant metastases was determined in 6 (16.6%) patients, mostly (n = 4) in lung and bone (4 lung and 2 bone metastasis). The 5- year multivariate survival analysis revealed the local control (Hazard ratio (HR = 6.9, P = 0.008) and stage of the tumor (HR = 3.21, P = 0.03) to have a statistical significant prognostic value (Table 5). 

**Table 4.** Overall Survival Rates in Advanced Stage Patients with Orbital Exenteration (P > 0.05).

Stage IV Patients (n = 28)	3- year Survival Rates (%,n/N)
**Orbital exenteration**	(25, 2/8)
**Orbital fat invasion (+)**	
**Orbita protected**	(37.5, 3/8)
**Orbital fat invasion (-)**	

**Table 5.** Cox Multivariate Analysis of Independent Predictors for Death from Sinonasal Malignancy

Predictors	Hazard Ratio (HR)	Confidence Interval (95%) (range)
**Local recurrence**	6.90	1.7 - 12.6
**Advanced Stage**	3.21	1.3 - 7.9
**Gender Male **	1.37	0.2 - 1.9
**Suprastructure area**	1.88	0.2 - 3.4
**Age (> 50), y**	1.22	0.2 - 1.9
**Epithelial forms**	1.57	0.1 - 2.7

## 5. Discussion

Sinonasal malignancies and less than 2% of all malignancies constitute about 1.5-3% of tumors in the upper respiratory tract (1). The most frequent location (50-80%) is the maxillary sinus (3). 26 (72.3%) of patients had maxillary sinus, in our study. The distribution by gender is 2:1 in favour of males (7,8). Male/ female ratio was 3 in our study group. Risk factors for sinonasal malignancy include exposure to industrial gases, nickel refining, , and leather painting. However, only one patient (2.7%) was worker of wood products and four patients (11.1%) were industry workers in our study group.

Sinonasal malignancies usually are presented with symptoms that are indistinguishable from rhinosinusitis, such as nasal obstruction, nasal discharge, epistaxis and facial pain (9). Early diagnosis is very important because in the advanced stage, the patients with tumor are not under control. For this reason, if sinusitis do not improve by medical therapy after two weeks, we suggest that repetition of nasal endoscopic examination and CT scan can diagnose the sinonasal malignancies at an early stage. Tumors of nasal cavities are usually diagnosed earlier, because of the obstructive symptoms and epistaxis (4).

Nine to twelve percent of patients are frequently asymptomatic. Some factors contribute to the delay in diagnoses, hence, the disease advances at the time of diagnosis.

Biopsy of the lesion is commonly performed using nasal endoscope in the office under topical or local anesthesia. Alternatively, the sampling can be performed in controlled environment of an operating room when a deep biopsy is required and bleeding occurs. The all sampling were performed using endoscope in operating room under local or topical anesthesia, in our study.

The most frequent histopathological types of sinonasal malignancies are squamous cell carcinoma followed by adenoid cystic carcinoma and adenocarcinoma (approximately 10% each) (6, 9). These results are similar in our study. Histopathology results in squamous cell carcinoma in 15 (41.7%) of our patients, too.

In previous studies, it was shown that the prognosis of sinonasal malignancies is poor, because the tumor is usually clinically advanced at the time of diagnosis (10, 11). The overall 3-year survival rates in cases with Stage 3 and Stage 4a were 46.7% and 30.8%, respectively in our study.

The incidence of metastatic lymph node involvement varies between 7% and 22 (the majority of series reported approximately 10% incidence of metastasis) (12-14). In previous studies, it was shown that the 5-year survival rate drops to 15 percent or below depending on the lymph node metastasis at the time of diagnosis (12-15). Some literature support the prophylactic central neck dissection in ganglion relapses occurring above 29% untreated N0 cases (9, 14).

It was reported that the location of tumor affects the prognosis (13, 16). Like most other publications, we found that 3-year overall survival rate was 66.7 % in patients with infrastructural localization while it was 25% in patients with supratructural localization in our study.

The condition of the surgical resection margin is another prognostic factor affecting the surgical treatment of sinonasal tumors. It has been demonstrated that effective local treatment is the main factor to improve survival rates (17). In our study, we found that patients with positive surgical margin had significantly worse survival rates than those with clear margin.

Despite the fact that long-term consequences are still anticipated, surgical options that can be offered according to oncologic principle give hope to patients with sinonasal malignity. Treatment strategies are essential for optimal surgical success and also local disease control. However, the primary problem in the treatment of the sinonasal malignancy has been the inability to achieve local control (14, 16). Overal 1- and 3-year survival rates was 45.5% and 27.3% in 22 patients with residue or recurrence of the local tumor, in our study. Local recurrences occurred in 16 patients with positive surgical margins and in six patients with free margins according to their own histopathological specimen results. It was shown that affected surgical margin significantly increases the risk of recurrence and correlates with poor prognosis in our study. This is the strong point of our study.

It was shown that neo-adjuvant chemotherapy (CT) could have better results in patients with locally advanced tumors arising in the paranasal sinuses. The patients in a previous study had an actual 5-year disease-free survival rate of 92% after neo-adjuvant chemotherapy at 55-month follow-up (18). In our study, there was no patient who had neo-adjuvant chemotherapy. Therefore, we couldn’t compare the results of surgery with neo-adjuvant chemotherapy and surgery without it, so this is the weak point of our study.

Many controversies exist over the protection of orbital contents during the surgical procedure of paranasal sinus cancers. Carrau et al. (19) found that 3-year survival rate was 52.0% in patients with exenterated orbit , whereas it was 59. 0 % in patient with preserved orbit in 58 cases of malignant sinonasal neoplasms with orbital expansion. There was no significant relationship between survival rates and type of surgery. According to this study, it was reported that involvement of the bones of the orbital walls is not an indication for orbital exenteration. Five- year overall survival was similar in both the exenterated orbit and preserved orbit. This finding was consistent with the literature. But, small number of patient in this group was other limitation of our study to make a clear statistical analysis in this issue.

As can be seen, sinonasal cancers have a poor survival rate by reason of advanced tumor and complex anatomy of the area. The major problem in the treatment of the sinonasal malignancy has been the inability to achieve local control. Local control of disease and survival are worse in patients who have histopathologically positive surgery margins. Local recurrence and tumor grade remain the main problem of treatment failure. The histological type, gender or age, do not contribute to prognosis in these patients. In addition, the role of orbital exenteration for increasing the survival rate is still controversial. Although our sample size is small, we demonstrated that the local control with orbital exenteration or others was the most important factor for optimal surgical results.
